# The efficiency of biologic therapy in a group of patients with rheumatoid arthritis

**Published:** 2015

**Authors:** BI Gavrilă, C Ciofu, V Stoica, E Panaitescu

**Affiliations:** * “Dr. I. Cantacuzino” Clinical Hospital, Bucharest, Department of Internal Medicine and Rheumatology, ”Carol Davila” University of Medicine and Pharmacy, Bucharest, Romania; ** Department of Medical Informatics and Biostatistics, ”Carol Davila” University of Medicine and Pharmacy, Bucharest, Romania

**Keywords:** rheumatoid arthritis, biologic therapy

## Abstract

**Objectives.** The following study aims to evaluate the monotherapy with biologic agents: Infliximab (IFX), Etanercept (ETA), Adalimumab (ADA) and Rituximab (RTX) in patients diagnosed with rheumatoid arthritis (RA).

**Methods.** To achieve these objectives, the database of “Dr. I. Cantacuzino” Clinical Hospital, Department of Internal Medicine and Rheumatology, was used.

The study was retrospective and descriptive, covering 168 patients with RA, followed for 12 months, from January 2012 to January 2013.

Admission criteria for the study were the following: patients diagnosed with RA according to ACR 1987/ EULAR 2010 criteria, disease activity score (DAS 28)> 5.1, positive inflammation tests, presence of RA refractory to classic remitting treatment administered at least 6 months prior to the initiation of biological therapy, on patients treated with RTX. They were considered non-responders after 6 months of treatment with anti tumor necrosis factor alpha (anti-TNF) and decided to switch agents with anti CD-20.

**Results** . Comparing values between any two points in time (baseline - 6 months -12 months) for any type of therapy, there were significant decreases in the values of erythrocyte sedimentation rate (ESR), reactive C protein (CRP) and disease activity score (DAS 28).

There were no significant differences between therapies regarding ESR at 6 months (p = 0.070, ANOVA) and 12 months (p = 0.375, Kruskal-Wallis), significant differences were regarding CRP at 6 and 12 months (p = 0.000, Kruskal-Wallis) and DAS 28 at 6 months (p = 0.000, Kruskal- Wallis) and 12 months (p = 0.018, Kruskal-Wallis).

**Conclusion** . All 4 therapies have proven efficient, prognostic markers decreasing gradually at 6 and 12 months.

**Abbreviations:** RA = rheumatoid arthritis, IFX = Infliximab, ETA = Etanercept, ADA = Adalimumab, RTX = Rituximab, ESR = erythrocyte sedimentation rate, CRP = reactive C protein, DAS 28 = disease activity score, anti TNF = inhibitor of tumor necrosis factor

## Introduction

Rheumatoid arthritis (RA) is a chronic inflammatory autoimmune disease with an incomplete elucidated etiology, presenting multiple extra-articular manifestations [**1**].

The disease may have an acute or insidious onset, and often progresses with major osteochondral lesions from the beginning. It has a prevalence of 1% within the general population and the severity of the disease is caused by various degrees of disability from the first two years.

The major impact on the body and the quality of life induces a higher level of morbidity and mortality, and therefore it is necessary to start an appropriate treatment as early as possible.

In addition to non-pharmacological therapies, therapeutic options in the treatment of AR are classic remitting drugs, biologic therapies, nonsteroidal anti-inflammatory drugs (NSAIDs) and steroids (glucocorticoids).

The basic treatment of RA includes disease-modifying antirheumatic drugs (DMARDs). This includes two major classes: synthetic compounds (sDMARDs) and biological agents (bDMARDs).

In 2013, Smolen JS et al. proposed a new classification, as it follows: the class sDMARDs contained synthetic conventional agents such as methotrexate, leflunomide, sulfasalazine, which were called csDMARDs, and a new agent sDMARDs - Tofacitinib (Janus Kinase inhibitor), which was included in the category of target agents - tsDMARDs.

The five inhibitors of the tumor necrosis factor (anti-TNF): Adalimumab, Etanercept, Infliximab, Golimumab, Certolizumab, the inhibitory agent to the co-stimulatory molecules (Abatacept), anti-CD20 (Rituximab), the inhibitors of interleukin-1 monoclonal antibodies (Anakinra) and interleukin 6 (Tocilizumab) were treated as original biological molecules - boDMARDs, while biosimilars recently approved by the European Medicines Agency (EMA) are included in the category of bsDMARDs [**2**,**3**].

Infliximab is a chimeric monoclonal antibody (murine and human) with a complex mechanism of action. It is administered as an infusion, having a dose of 3-5 to 10 mg/ kg body weight. This agents’ infusions induction is performed at weeks 0, 2 and 6, and thereafter the treatment is administered by infusion at every 8 weeks [**4**].

Etanercept, an antibody that acts in the soluble receptors of TNF, is administered as a subcutaneous injection; phials of 25 mg are administered 2 times/ week and 50 mg phials once a week [**5**].

Adalimumab, a human anti TNF inhibitor is administered subcutaneously, 40 mg at every 2 weeks. Favorable effects were demonstrated both cutaneous and articular [**6**].

Rituximab is a chimeric anti-lymphocyte monoclonal antibody B/ CD-20. 1,000 mg i.v. are administered at 2 weeks (2 x 500 mg), followed by a further cure to 6 months. This therapy is used in patients who have not responded to anti-TNF therapy or for patients with contraindications to treatment with anti-TNF therapy (e.g. various neoplastic diseases) [**7**,**8**].

## Objectives

The objective of the study was to evaluate the efficacy of biological agents: Infliximab, Etanercept, Adalimumab and Rituximab in patients diagnosed with severe and active RA.

The main parameters used to assess the efficacy of the treatment were the following: DAS 28 score, ESR and CRP. These markers were determined three times during the study: baseline, at six months and at twelve months.

## Methods

To achieve these objectives, the database of “Dr. I. Cantacuzino” Clinical Hospital, Department of Internal Medicine and Rheumatology, was used.

The study was retrospective, descriptive and included 168 patients with RA, followed for 12 months, from January 2012 to January 2013.

The inclusion criteria for the study were the following:

• patients who were diagnosed with RA according to ACR 1987/ EULAR 2010 criteria [**9**,**10**],

• with DAS 28 > 5.1,

• positive inflammation tests: CRP> 5 mg/ dl, ESR> 15 mm/ h,

• presence of RA refractory to classic remitting treatment administered at least 6 months prior to the initiation of biological therapy (maximum dose possible of two drugs, one should be Methotrexate unless contraindicated/ intolerance).

• written consent for the administration of the biological treatment,

• on patients treated with Rituximab, they were considered non-responders after 6 months of treatment with anti TNF and decided to switch agents with anti CD-20.

All the patients were clinically and laboratory assessed before the use of biological treatment and then at every 6 and 12 months. Each patient was assessed by the treating physician, having the possibility to change the therapy if clinical and laboratory parameters were not improved as recommended by EULAR [**11**].

**Statistical analysis**

Variations of DAS 28 score, ESR, CRP were expressed as mean ± SD or median and quartile 25% respectively 75%. When comparing values between different time groups (0, 6, 12 months) the Student test was used in pairs with Bonferroni correction.

For comparison, ANOVA or Kruskall Wallis tests were used depending on data distribution and the multiple comparison tests.

For the statistical analysis software package, SPSS version 15.0, was used.

Statistical tests were considered significant at p ≤ 0.05.

## Results

168 patients were included in the study, 130 women (77.38%) and 38 men (22.62%).

Among the patients with RA, 8 (4.76%) were < 35 years, 60 (35.71%) were aged between 35 and 55 years, and 100 patients (59.52%) had > 55 years.

**Table 1:** Distribution groups according to sex and age

	Patients		Age		
Gender	Male	Female	< 35	35 – 55	> 55
No.	38	130	8	60	100
%	22,62	77,38	4,76	35,71	59,52

The repartition of patients according to medication: 25 (14.88%) patients were treated with IFX, 18 (10.71%) with ADA, 34 (20.24%) with ETA and 91 (54.17%) with RTX.

**Fig. 1:**
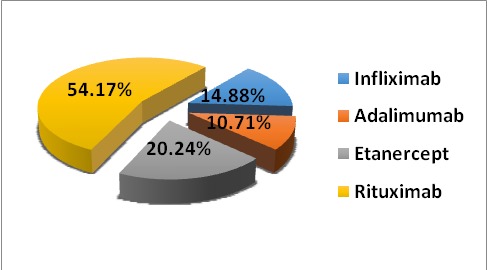
Distribution of patients with RA according to the treatment

**The evolution of ESR, CRP and DAS 28 in patients treated with Infliximab**

Patients selected for the study had initial values of ESR >15 mm/ 1h: 19 patients (76%) had ESR values between 15-35 mm/ 1h, and 6 patients (24%) had values >35 mm/ 1h.

At 6 months, the values for ESR were the following: 8 patients had ESR values <15mm/ 1h, 16 patients had values between 15-35mm/ 1h and 1 patient had ESR values of >35 mm/ 1h.

At 12 months, ESR values were the following: 24 patients <15 mm/ 1h and 1 patient was between 15-35mm/ 1h. No other patients had ESR values of >35 mm/ 1h.

The baseline levels of reactive C protein were the following: between 5-15 mg/ dl for 23 patients (92%) and for 2 patients (8%) 15> mg/ dl.

After 6 months of treatment, CRP values were <5 mg/ dl for 16 patients (64%), between 5-15 mg/ dl for 8 patients (32%), and only 1 patient (4%) had CRP of >15 mg/ dl.

After 12 months of treatment with Infliximab, the values for CRP were < 5mg/ dl for 24 patients (96%) and 1 patient (4%) had values between 5-15 mg/ dl. No other patients had CRP values of >15 mg/ dl.

**Fig. 2:**
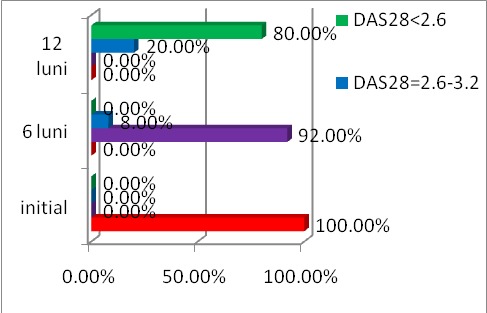
The evolution of DAS 28 for 12 months in patients with RA treated with Infliximab

The evolution of DAS 28 score under treatment with IFX is shown in **Fig. 2**.

**The evolution of ESR, CRP and DAS 28 in patients treated with Etanercept**

Values for ESR were between 15-35 mm/ 1h for 27 patients (79.41%) and >35 mm/ 1h for 7 patients (20.59%).

At 6 months, the values for ESR were <15 mm/ 1h for 14 patients (41.18%), between 15-35 mm/ 1h for 18 patients (52.94%) and >35 mm/ 1h for 2 patients (5.88%).

At 12 months, values for ESR were <15 mm/ 1h for 31 patients (91.18%) and between 15-35 mm/ 1h for 3 patients (8.82%). No other patients had ESR values of >35 mm/ 1h.

The initial value of the CRP in patients with RA treated with ETA: 5-15 mg/ dl in 29 cases (85.30%) and > 15 mg/ dL in 5 cases (14.70%).

The evaluation at 6 months after the initiation of therapy: 22 patients (64.70%) had CRP values of <5 mg/ dl, 11 patients (32.35%) had CRP in the range of 5-15 mg/ dl and only in 1 patient (2.95%) a CRP value of > 15 mg/ dl was detected.

The final assessment at 12 months after the initiation of treatment revealed CRP of <5 mg/ dl in 32 cases (94.12%) and CRP values between 5-15 mg/ dl in other 2 patients (5.88%).

**Fig. 3:**
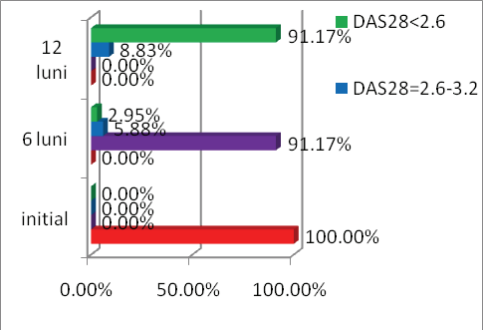
The evolution of DAS 28 for 12 months in patients with RA treated with Etanercept

The evolution of DAS 28 score under the treatment with ETA is shown in **Fig. 3**.

**The evolution of ESR, CRP and DAS 28 in patients treated with Adalimumab**

Baseline value for Adalimumab group for ESR was between 15-35 mm/ 1h for 6 patients (33.33%) and >35 mm/ 1h for 12 patients (66.67%).

At 6 months, the values for ESR were <15 mm/ 1h for 4 patients (22.22%), between 15-35 mm/ 1h for 11 patients (61.11%) and >35 mm/ 1h for only 3 patients (16.67%).

The final analysis performed after 12 months of therapy with Adalimumab detected 16 patients (88.89%) with ESR values within the physiological limits (<15 mm/ 1h) and 2 patients (11.11%) with ESR between 15-35 mm/ 1h.

The baseline value for CRP was between 5-15 mg/ dl for 9 patients (50%) and >15 mg/ dl for 9 patients (50%). No patients with CRP value of <5 mg/ dl were included in the study.

At 6 months, the values for CRP were <5 mg/ dl for 2 patients (11.11%), between 5-15 mg/ dl for 13 patients (72.22%) and >15 mg/ dl for only 3 patients (16.67%).

At 12 months, the values for CRP were <5 mg/ dl for 16 patients (88.89%), therefore in physiological limits and between 5-15 mg/ dl for 2 patients (11.11%).

**Fig. 4:**
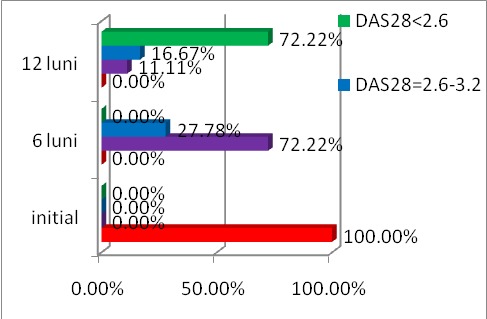
The evolution of DAS 28 for 12 months in patients with RA treated with Adalimumab

The evolution of DAS 28 score under treatment with ADA is shown in **Fig. 4**.

**The evolution of ESR, CRP and DAS 28 in patients treated with Rituximab**

Baseline value for ESR was between 15-35 mm/ 1h for 62 patients (68.13%) and >35 mm/ 1h for 29 patients (31.87%).

Inflammatory samples taken at 6 months after the initiation of therapy revealed 27 patients (29.67%) with ESR <15 mm/ 1h, 59 patients (64.84%) with ESR values between 15-35 mm/ 1h and 5 patients (5.49%) with ESR > 35 mm/ 1h.

ESR analysis at 12 months after the initiation of therapy revealed the following results: 70 patients (76.92%) with ESR <15 mm/ 1h and 21 patients (23.08%) with values between 15-35 mm/ 1h. No patient had values of ESR> 35 mm/ 1h.

Baseline values of CRP were between 5-15 mg/ dl in 63 cases (69.23%) and >15 mg/ dl in 28 patients (30.77%).

At 6 months after the beginning of treatment, the samples showed values of CRP <5 mg/ dl in 26 cases (28.57%), between 5-15 mg/ dl in 55 cases (60.44%), 10 patients with CRP of >15 mg/ dl (10.99%).

At 12 months it was found that 73 patients (80.22%) had CRP values of <5 mg/ dl, 13 patients (14.29%) had CRP in the range of 5-15 mg/ dl, 5 patients (5.49%) had values of >15 mg/ dl.

**Fig. 5:**
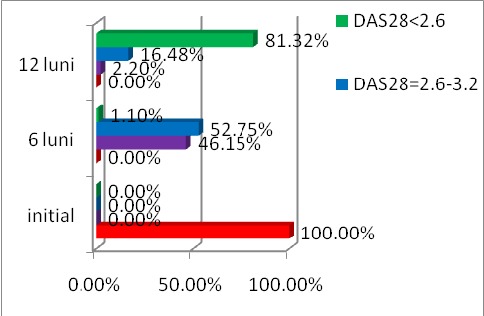
The evolution of DAS 28 for 12 months in patients with RA treated with Rituximab

The evolution of DAS 28 score under treatment with RTX is shown in **Fig. 5**.

Comparing the values of the parameters analyzed anytime between 0-6 months, 0-12 months or 6-12 months, the patients have achieved significant improvements over previous values assessments for ESR, CRP and DAS 28.

At 6 months, the average ESR value was 16.68 ± 6,19 for Infliximab, Etanercept 16.32 ± 6.98, Adalimumab 21.89 ± 9.32 and 18.10 ± 7.72 for Rituximab without noticing significant differences between therapies (ANOVA 0.070).

**Table 2:** The evolution of ESR, CRP and DAS 28 for 12 months based on the average (Mean ± SD)

	**Infliximab**	**Etanercept**	**Adalimumab**	**Rituximab**
Nr	**25**	**34**	**18**	**91**
VSH_init	33,08 ± 18,12	32,59 ± 18,07	48,67 ± 22,96	33,80 ± 14,66
VSH_6l	16.68±6.19	16.32±6.98	21.89±9.32	18.10±7.72
VSH_12l	9.76±4.10	9.82±4.45	11.50±3.65	10.85±5.53
CRP_init	11.44±10.69	10.25±+5.64	23.36±23.94	17.00±16.00
CRP_6l	5.92±6.16	4.85±2.48	10.33±11.00	8.63±8.23
CRP_12l	2.68±2.72	2.19±1.53	3.34+1.68	4.66±4.42
DAS28_init	5.72±0.32	5.72±0.40	5.69±0.44	5.60±0.30
DAS28_6l	3.61±0.28	3.70±0.41	3.85±0.73	3.27±0.37
DAS28_12l	2.35±0.33	1.97±0.53	2.35±0.73	2.08±0.61

**Table 3:** The evolution of ESR, CRP and DAS 28 for 12 months according to the median [25%; 75%]

	**Infliximab**	**Etanercept**	**Adalimumab**	**Rituximab**
No	**25**	**34**	**18**	**91**
VSH_init	28.00 [22.00, 34.00]	27.00 [20.00, 35.00]	39.00 [33.00, 68.25]	29.00 [25.00, 38.00]
VSH_6l	15.00 [13.50, 18.00]	16.00 [11.00, 18.00]	18.50 [14.75, 29.00]	16.00 [13.00, 20.00]
VSH_12l	9.00 [7.00, 12.50]	9.00 [6.75, 13.00]	12.00 [9.0, 14.0]	10.00 [7.00, 14.00]
CRP_init	8.65 [7.84, 10.78]	8.24 [7.34, 10.9300]	16.020 [10.65, 24.19]	10.20 [8.30, 17.45]
CRP_6l	4.61 [3.58, 5.59]	4.48 [3.27, 5.33]	6.825 [5.44, 10.53]	5.67 [4.68, 8.45]
CRP_12l	1.96 [1.13, 3.68]	1.93 [0.87, 3.05]	3.39 [1.78, 4.38]	3.70 [2.12, 4.85]
DAS28_init	5.66 [5.54, 5.91]	5.61 [5.38, 5.99]	5.52 [5.33, 6.07]	5.57 [5.38, 5.72]
DAS28_6l	5.00 [4.82, 5.21]	3.76 [3.45, 3.99]	3.66 [3.17, 4.74]	3.19 [3.01, 3.58]
DAS28_12l	3.63 [3.41, 3.82]	1.83 [1.54, 2.43]	2.23 [1.74, 2.74]	2.02 [1.64, 2.46]

Regarding CRP at 6 months, the values were 4.61 [3.58, 5.59] for the group under treatment with Infliximab, 4.48 [3.27, 5.3] for Etanercept, 6.825 [5.44, 10.53] for Adalimumab and 5.67 [4.68, 8.45] for Rituximab, with significant differences between therapies (p=0.000, Kruskal-Wallis) as it follows: Infliximab - Adalimumab (p = 0.003), Infliximab - Rituximab (p = 0.026), Etanercept - Adalimumab (p = 0.000), Etanercept - Rituximab (p = 0.000).

Median values for DAS 28 at 6 months were the following: 5.00 [4.82, 5.21] for the Infliximab group, 3.76 [3.16, 3.99] for Etanercept, 3.66 [3.17, 4.74] for Adalimumab group and 3.19 [3.01, 3.58] for Rituximab. There were significant differences between therapies (p=0.000, Kruskal-Wallis), as it follows: Infliximab - Rituximab (p = 0.000), Etanercept - Rituximab (p = 0.000), Adalimumab - Rituximab (p = 0.002).

Regarding ESR at 12 months, there were no significant differences between therapies (p=0.375, Kruskal-Wallis).

On the other hand, there were significant differences between therapies at 12 months regarding CRP (p=0.000, Kruskal-Wallis), as it follows: Infliximab - Rituximab (p = 0.010) and Etanercept - Rituximab (p = 0.000); values for each group: 1.96 [1.13, 3.68] for Infliximab, 1.93 [0.87, 3.05] for Etanercept, 3.39 [1.78, 4.38] for Adalimumab and 3.70 [2.12, 4.85] for patients treated with Rituximab.

For DAS 28 – the 12 months comparison revealed significant differences between groups (p=0.018, Kruskal-Wallis): Infliximab - Etanercept (p = 0.022); values for each group were 3.63 [3.41, 3.82] for Infliximab, and 1.83 [1.54, 2.43] in the group of patients treated with Etanercept.

## Conclusions

All 4 therapies have proven efficient, prognostic markers decreasing progressively at 6 and 12 months.

Regarding ESR, there were no significant differences between groups at any time of the tests.

Following the evolution of CRP, there were significant differences between the groups of patients at 6 months; the best response rate was seen in patients treated with Infliximab and Etanercept. The lowest response rate for CRP at 12 months was found in patients treated with Rituximab.

Regarding DAS 28, the clinical remission score, the best response was in the group of patients treated with Etanercept.

**Conflicts of interest:** None declared.

**Acknowledgments:** This work received financial support through the project entitled “CERO – Career profile: Romanian Researcher” grant number POSDRU/159/1.5/S/135760, co-financed by the European Social Fund for Sectoral Operational Programme Human Resources Development 2007-2013.

I would like also to thank physicians from “Dr. I. Canatcuzino” Clinical Hospital, Bucharest, Clinic of Internal Medicine and Rheumatology, for providing patients.
